# Supplementary material for: Structural genomics applied to the rust fungus Melampsora larici-populina reveals two candidate effector proteins adopting cystine knot and NTF2-like protein folds
Source: Sci Rep. 2019 Dec 2;9:18084. doi: 10.1038/s41598-019-53816-9 (PMC6889267; doi:10.1038/s41598-019-53816-9)
Supplement: Supplementary file 1 — Supplementary information [file 41598_2019_53816_MOESM1_ESM.pdf]

Supplementary tables and figures

Structural genomics applied to the rust fungus *Melampsora larici-populina* reveals two candidate effector proteins adopting cystine knot and NTF2-like protein folds

Karine de Guillen[1], Cécile Lorrain[2], Pascale Tsan[3], Philippe Barthe[1], Benjamin Petre[2], Natalya Saveleva[2], Nicolas Rouhier[2], Sébastien Duplessis[2], André Padilla[1] and Arnaud Hecker[2,*]

[1]Centre de Biochimie Structurale (CBS), INSERM U1054, CNRS UMR 5048, Univ Montpellier, F-34090 Montpellier, France.

[2]Université de Lorraine, INRA, IAM, F-54000 Nancy, France.

[3]Université de Lorraine, CNRS, CRM2, F-54000 Nancy, France.

* Corresponding author: arnaud.hecker@univ-lorraine.fr

Table S1: Primers used for PCR cloning in pET expression vectors

Protein ID	Cloning Enzymes	Forward Primer	Reverse Primer	Expression vector
MLP124478-$(His)_6$	*Nde*I/*Xho*I	5'-ggggggCATATGAAAGGTCGACACAAAAATG	5'-ggggggCTCGAGACATGTAACTTTCACGTTCC	pET-26b
MLP124530-$(His)_6$	*Nde*I/*Xho*I	5'-ggggggCATATGAAAGACATCAATTTAGTTG	5'-ggggggCTCGAGTTCTGCAGACTCATTTTTGAAG	pET-26b
MLP124111-$(His)_6$	*Nde*I/*Xho*I	5'-ggggggCATATGCAAACTCCCACCGGAGG	5'-ggggggCTCGAGGGGTACAACACATTTGTAACAAG	pET-26b
MLP124561-$(His)_6$	*Nde*I/*Xho*I	5'-ggggggCATATGGCAGACTGGCACACAGCAG	5'-ggggggCTCGAGATAATCCCTCGTTATGACCAAAG	pET-26b
MLP37347-$(His)_6$	*Nde*I/*Xho*I	5'-ggggggCATATGGTAGAACCACTCCCACTCG	5'-ggggggCTCGAGCCACTTCTTGGGTTTTGGTAC	pET-26b
MLP109567-$(His)_6$	*Nde*I/*Xho*I	5'-ggggggCATATGGGACTGGTTTCTAGCTTAGC	5'-ggggggCTCGAGGTGAACCTCTTGCTCGGGAC	pET-26b
MLP124017-$(His)_6$	*Nco*I/*Xho*I	5'-ggggggCCATGGAGCTACCAGAAAGTTTTG	5'-ggggggCTCGAGGTCTATGGCAACATATGGTGC	pET-28a
MLP107772-$(His)_6$	*Nde*I/*Xho*I	5'-ggggggCATATGCAGGgtcgaGCAATCTGTC	5'-ggggggCTCGAGAGGACAATTTGGTCCGTTTCC	pET-26b
MLP124202-$(His)_6$	*Nde*I/*Xho*I	5'-ggggggCATATGAATTTCTGTGTTAAGGGAC	5'-ggggggCTCGAGTTTGTGAGATGTCTTTCTTCC	pET-26b
MLP124266-$(His)_6$	*Nde*I/*Not*I	5'-ggggggCATATGTGTGAATTTATAGAGGATTCAG	5'-ggggggGCGGCCGCTTAGTGATGGTGATGGTGATGTAGAG AGCTCAATACACATACACC	pET-26b
MLP124499-$(His)_6$	*Nde*I/*Not*I	5'-ggggggCATATGACTCACAGTGTCAAGAGCAAAG	5'-ggggggGCGGCCGCTCAGTGATGGTGATGGTGATGAGAAT CCGCGCGGTAGTGATAG	pET-26b

Table S2: Statistics for 20 NMR structures of MLP124266

	MLP124266
NOE restraints	653
Short range ($\lvert i-j \rvert \leq 1$)	510
Medium range ($1< \lvert i-j \rvert <5$)	102
Long range ($\lvert i-j \rvert \geq 5$)	41
H-bond restraints	7
Dihedral restraints [a]	75
Number of NOE violations	
> 0.0 Å	82
> 0.3 Å	45
> 0.5 Å	18
Dihedral violations	
> 0°	63
> 5°	23
Ramachandran plot statistics	
most favourable regions (%)	81.9
allowed regions (%)	15.3
disallowed regions (%)	2.8
RMSD (Å) [b]	
Backbone	1.44 ± 0.52
Heavy atoms	2.08 ± 0.53

[a] Dihedral restraints were generated using DANGLE.
[b] RMSD were calculated over residues 39 to 69.

Table S3: NMR experiments acquired for structure calculations and chemical shift assignments for MLP124017

		Size			Sweep width (ppm)						
Experiments	nuclei	F3	F2	F1	F3	F2	F1	Mix(ms)	NS	D1(s)	B_o(MHz)
^{15}N-HSQC	^{1}H, ^{15}N	1800	180	-	13.94	36	-	-	4	1.0	800
HNCO(*)	^{1}H,^{15}N,^{13}C	1366	60	100	13.94	36	15	-	8	0.2	800
HNCA(*)	^{1}H,^{15}N,^{13}C	1362	50	60	13.94	36	30	-	16	0.2	800
HNCOCACB(*)	^{1}H,^{15}N,^{13}C	1366	60	100	13.94	36	70	-	32	0.2	800
HNCACO(**)	^{1}H,^{15}N,^{13}C	1366	40	100	13.94	36	15	-	16	1.0	800
HNCACB(*)	^{1}H,^{15}N,^{13}C	1362	50	60	13.94	36	60	-	32	0.2	800
^{15}N-NOESY-HSQC	^{1}H,^{15}N, ^{1}H	1800	64	360	13.94	25	13.94	150	8	1	800
^{15}N-TOCSY-HSQC	^{1}H,^{15}N, ^{1}H	1800	64	360	13.94	25	13.94	54	8	1	800
NOESY (D_2O)	^{1}H, ^{1}H	2048	512	-	12.03	12.03	-	150	128	1	700
TOCSY (D_2O)	^{1}H, ^{1}H	2048	512	-	12.03	12.03	-	57.6	128	1	700
^{15}N-HSQC (***)	^{1}H, ^{15}N	1112	256	-	15.95	36	-	-	2	0.2	700

Experiments were recorded using the TOPSPIN Library (v. 2.1) at 298 K.
(*) BEST pulse sequences
(**) Watergate version
(***) SOFAST version (from Institut de Biologie Structurale, Grenoble, http://www.ibs.fr/research/scientific-output/software/pulse-sequence-tools/article/ibs-pulse-sequence-tools-for-bruker-spectrometers)

Table S4: Statistics for 20 NMR structures of MLP124017

	MLP124017
NOE restraints	1727
Short range ($\lvert i-j \rvert \leq 1$)	999
Medium range ($1 < \lvert i-j \rvert < 5$)	345
Long range ($\lvert i-j \rvert \geq 5$)	383
H-bond restraints	102
Dihedral restraints [a]	214
Number of NOE violations	
> 0.0 Å	186.35
> 0.3 Å	0.05
> 0.5 Å	0
Dihedral violations	
> 0°	22.25
> 5°	0
Ramachandran plot statistics	
Most favourable regions (%)	89.8
Additionally allowed regions (%)	9.8
Generously allowed regions (%)	0.4
Disallowed regions (%)	0.1
RMSD (Å) [b]	
Backbone	1.24 ± 0.39
Heavy atoms	1.81 ± 0.40

[a] Residues in regular secondary structures were derived from the chemical shifts using TALOS+ software.
[b] Main chain atoms (N, Cα, C) over the residues 20-81 and 100-141.

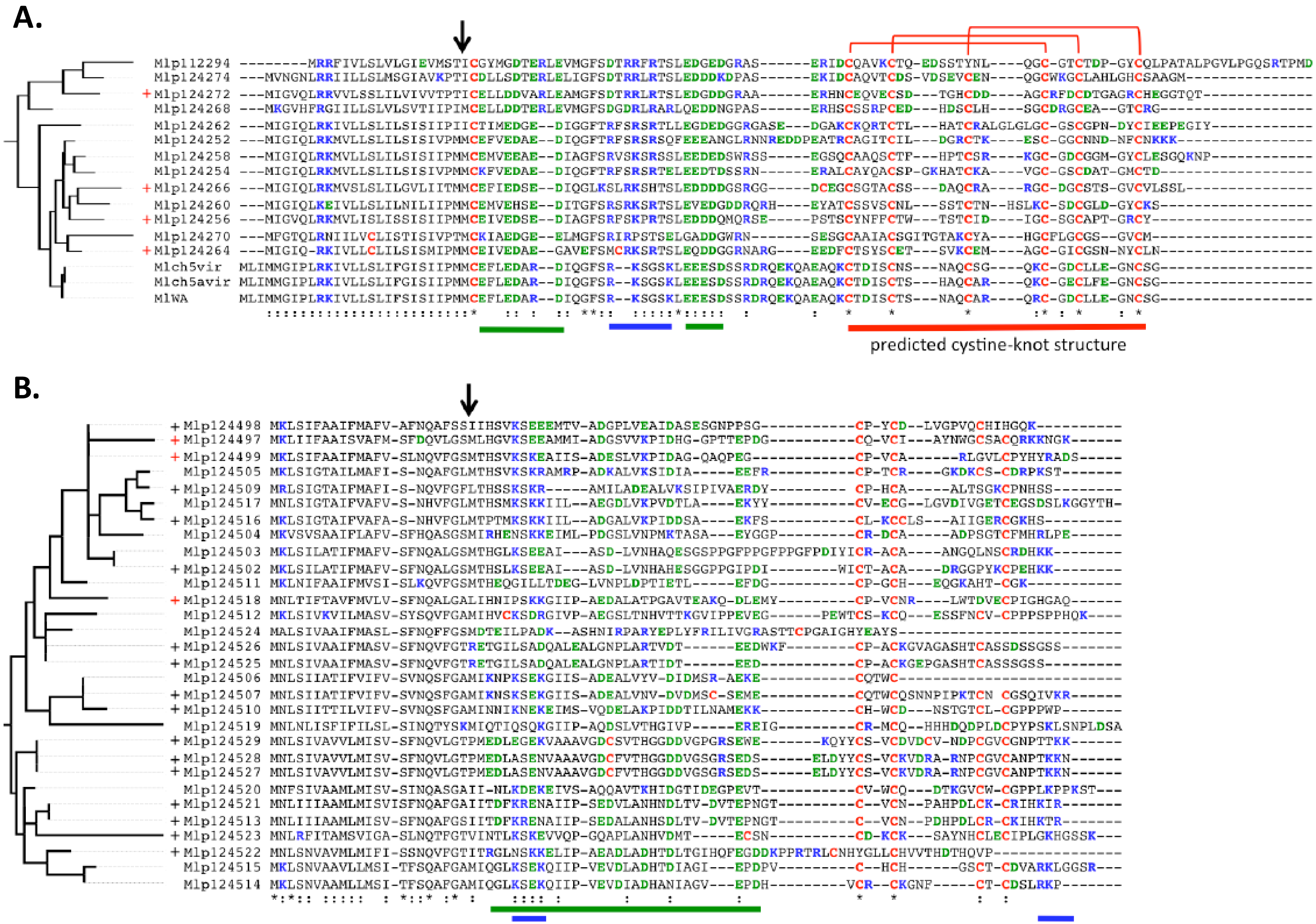
A.
Mlp112294
Mlp124274
Mlp124272
Mlp124268
Mlp124262
Mlp124252
Mlp124258
Mlp124254
Mlp124266
Mlp124260
Mlp124256
Mlp124270
Mlp124264
Mlch5vir
Mlch5avir
MlWA
predicted cystine-knot structure
B.
Mlp124498
Mlp124497
Mlp124499
Mlp124505
Mlp124509
Mlp124517
Mlp124516
Mlp124504
Mlp124503
Mlp124502
Mlp124511
Mlp124518
Mlp124512
Mlp124524
Mlp124526
Mlp124525
Mlp124506
Mlp124507
Mlp124510
Mlp124519
Mlp124529
Mlp124528
Mlp124527
Mlp124520
Mlp124521
Mlp124513
Mlp124523
Mlp124522
Mlp124515
Mlp124514

Figure S1: Sequence characteristics of proteins from MLP124266 and MLP124499 families

Phylogenetic trees were built from corresponding aligned protein sequences from MLP124266 (**A.**) and MLP124499 (**B.**) families. Branch length is proportional to phylogenetic distance. Black '+' indicates that expression of the gene has been detected *in planta* [1,2], red '+' indicates that the RT-qPCR expression profile of the gene has been established [3]. The black arrows indicate the predicted cleavage site of the signal peptide. Cysteine residues are in red, basic residues (lysine/K and arginine/R) are in blue, acidic residues (aspartic acid/D and glutamic acid/E) are in green. Blue and green lines show basic and acidic amino acid stretches and the red line delimited the predicted cystine knot motif. An amino acid conservation of 50% or more is marked by a colon. An amino acid conservation of 90% or more is marked by a star.

A.

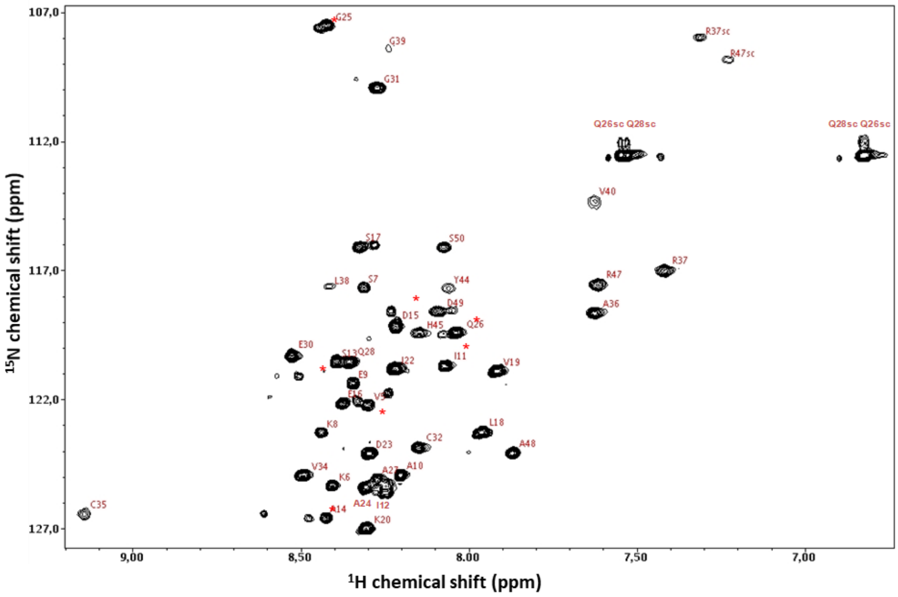

B.

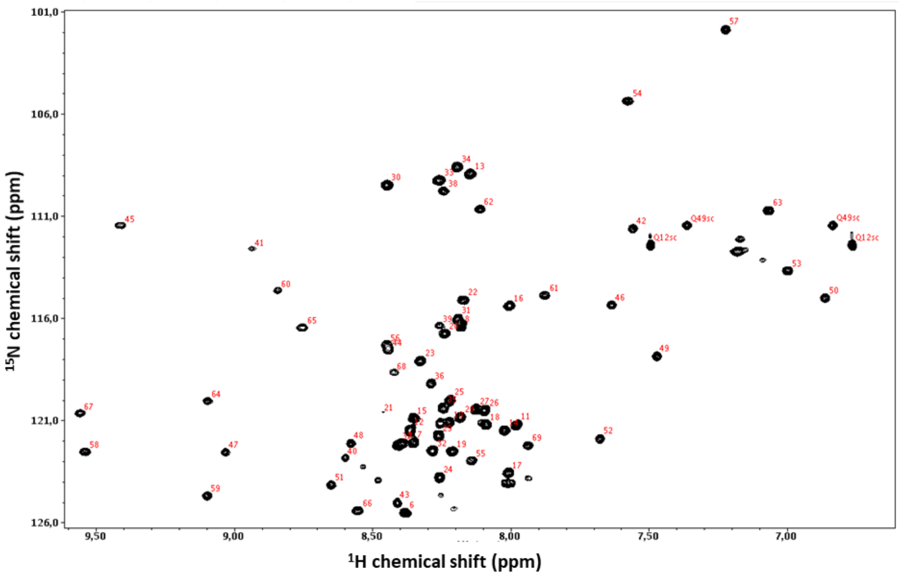

Figure S2: ^{1}H-^{15}N HSQC spectra of recombinant MLP124499 and MLP124266.

The NMR experiment was recorded on a 600 MHz spectrometer, at 298 K, on a uniformly 15N-labelled sample in phosphate buffer pH 6.0. Backbone amide signals of MLP124499 (**A**) or MLP124266 (**B**) are labelled with the residue number and « sc » refers to side-chain amide signals. Minor peaks observed in MLP124499 are indicated by asterisks (*).

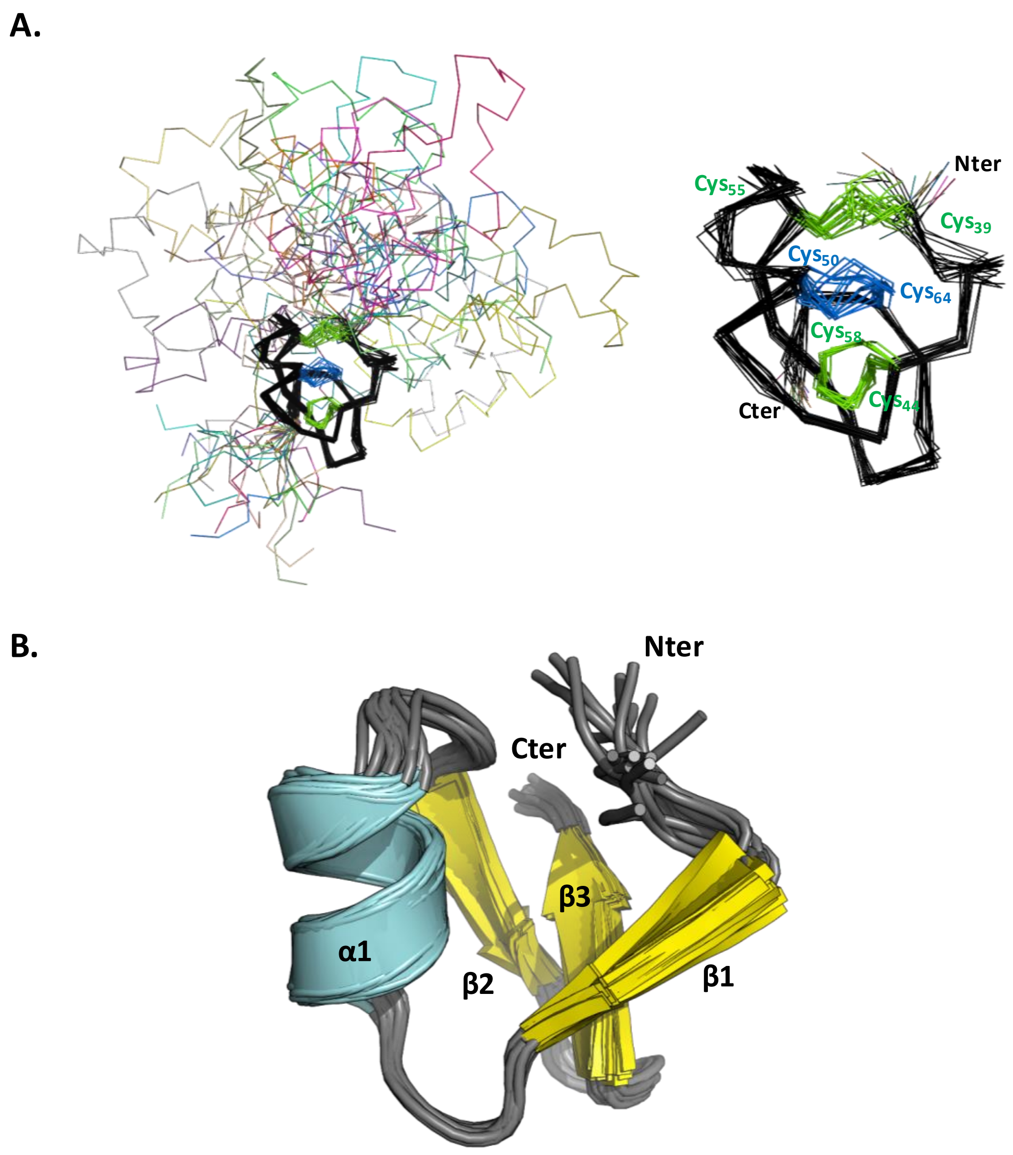

Figure S3: Superposition of the 20 lowest-energy NMR structures of MLP124266.

A. Superimposition of backbone heavy atoms (N, Cα and C) for a family of 20 lowest energy NMR structures (left) and zoom on cystine knot (right). Each structure is rendered as ribbon. Cystine knot (region 39-65) is coloured in black and the three disulfide bridges are labelled and coloured in green (Cys_{39}-Cys_{55}, Cys_{44}-Cys_{58}) or in blue (Cys_{50}-Cys_{64}). Nter and Cter extremities of the cystine knot are indicated.

B. NMR models were aligned by their cystine knot region (region 39-65). Secondary structures are labelled and represented as cartoon. The three disulfide bridges are not represented for clarity of the figure. Nter and Cter extremities of the cystine knot region are indicated.

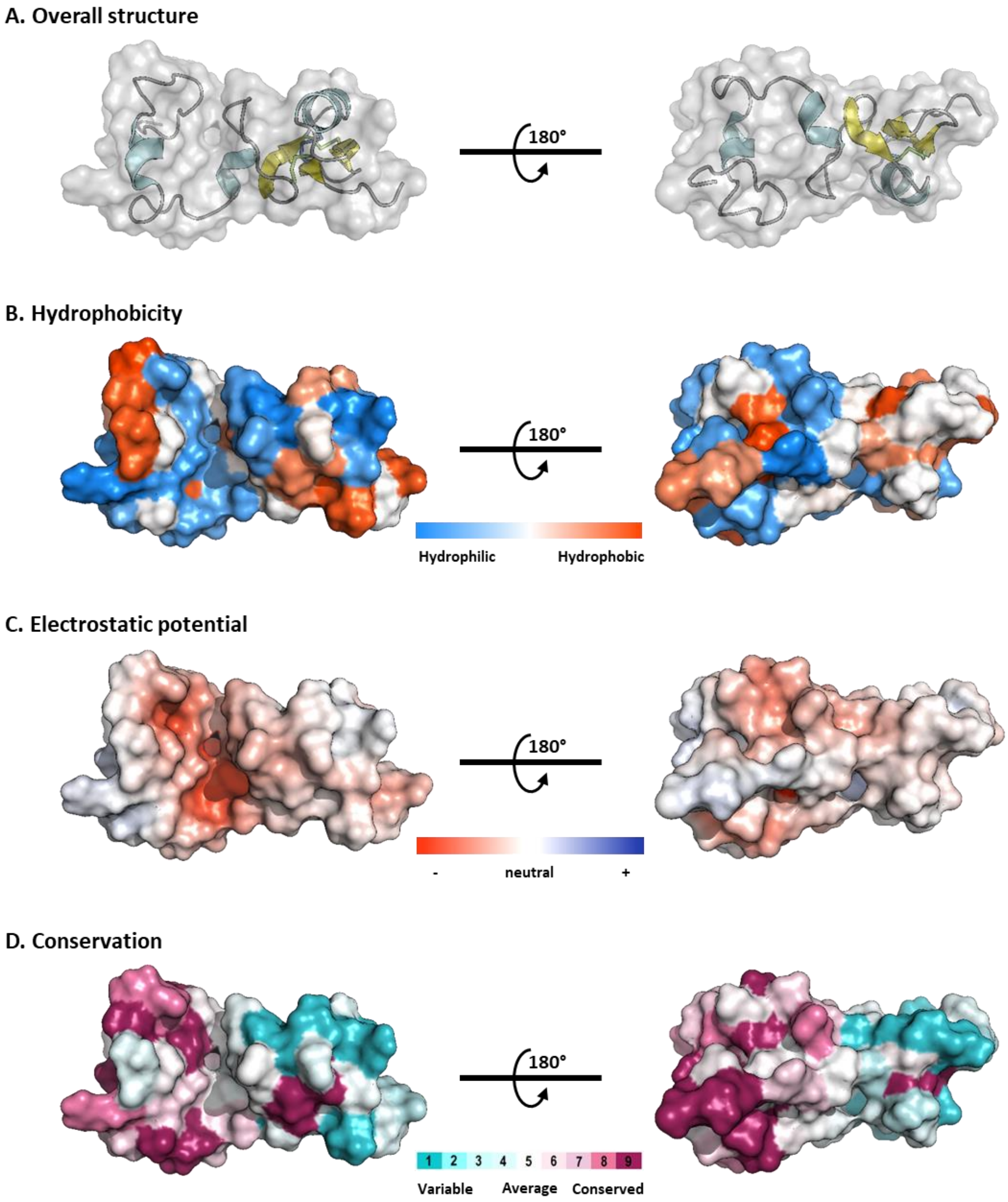

Figure S4: Biophysical properties of MLP124266.

Front and rear views on the surface of MLP124266 (**A**) illustrating hydrophobic potential (**B**), the electrostatic Coulomb potential at pH 7.0 using APBS plugin from Pymol 2.0 software with a contour of -10 kT/e to 10 kT/e (**C**) and the conservation of residues generated by Consurf server (**D**).

Hydrophobic and hydrophilic patches are shown in red and in blue, positive-charge and negative-charge densities are coloured in blue and red respectively. Conservation scale ranged from high (purple) to poor conservation (light blue).

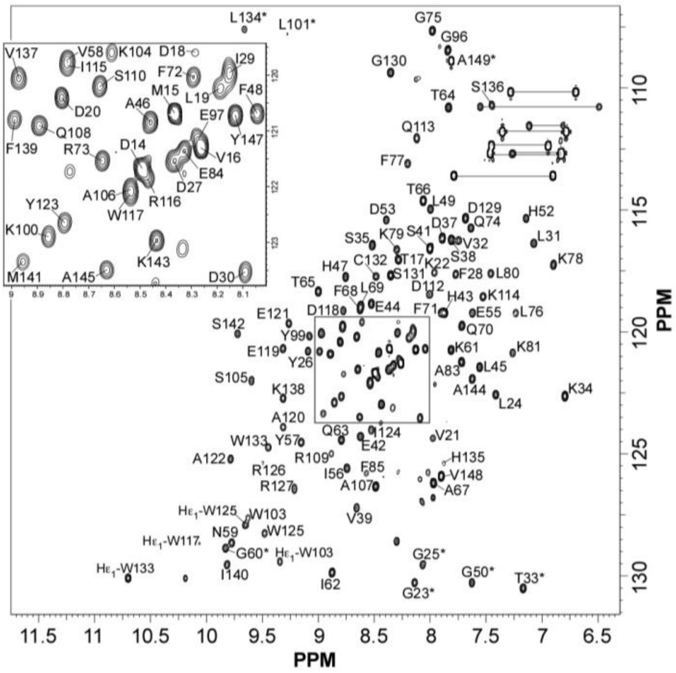

Figure S5: ^{15}N-HSQC spectra of MLP124017.

Cross peak assignments are indicated using the one-letter amino acid and number (the asterisk indicates a folded peak). The central part of the spectrum is expanded in the insert. Missing or non-assigned residues: 1-15, 40, 82, 86-95, 98, 102, 111, 128, 150, 151.

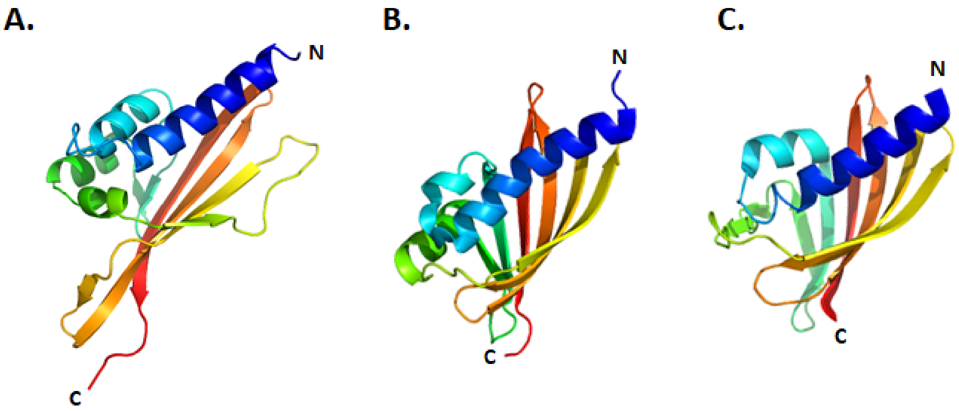

D.

```
                   :         :         :         :         |         :         :
MLP124017 DMVTDLDVKGLGYDFIDLVTKSPDSVNSEHELAHFLGPHDPEIYVNGKIQTTTAFLQFFRQGLFKKLKDA
3BLZ-J    -NTTYVQEYHAIVEVLSKYNE----KADSTIXRPAFS-SQATIFGVLTGGPIQGLFDVIDNVFHPSP--A
3FKA-C    --XTTSEHIAALTALVETYVX-------DRPALERIFFGKASEVGHLLWNSRDAFIAXCEDAADAET--P
                   :         :         :         :         |         :         :
MLP124017 LLLHHHHHHHHHHHHHHHHHLLLLLHHHHHHHHHHHLLLLLEEEELLEEEELHHHHHHHHHHHHLLLLLL
3BLZ-J    -LLLHHHHHHHHHHHHHHHHH----LLLHHHHHHHEE-EEEEEEEEEEEEELHHHHHHHHHLLLLLL--L
3FKA-C    --LLHHHHHHHHHHHHHHHHH-------LHHHHHHHEEEEEEEEEEEEEEEHHHHHHHHHHHLLLLL--L

                   :         :        100        :         :
MLP124017 EFAINVSGKVKEGEGYKLVWKSAAQRSHDQKIRWDEAEAYIWRRKDGSCWLHSVKFIM
3BLZ-J    KAA--ARIDIVGTAASARIDTDD----------FRFTDFFLLKV---GKWTVSKIYHT
3FKA-C    FWA--ISSVSVQGIAXLHVNDWAG---------XRFDDLTVLLH---GSWRISKVYRI
                   :         :        100        :         :
MLP124017 LLLLLLLLLLLLLLLEEEEEELLLLLLLLLLLLLLEEEEEELLLLLLLLLLLEEEEEL
3BLZ-J    EEE--EEEEEELLEEEEEEEEEE----------EEEEEEEEEEE---LEEEEEEEEEE
3FKA-C    LEE--EEEEEEELEEEEEEEEELL---------EEEEEEEEEEE---LEEEEEEEEEE
```

Figure S6: MLP124017 and two bacterial proteins SBAL_0622 and SPO1084 have similar Nuclear-transport factor 2-like fold.

Structures of (**A**) MLP124017, (**B**) SBAL_0622 (PDB code: 3BLZ) and (**C**) SPO1084 (PDB code: 3FKA) were represented in cartoon with a rainbow coloration using PYMOL (http://www.pymol.org). (**D**) Multiple structural alignment for the three proteins was done with Dali [4] with the MLP124017 as the reference for numbering. The first part shows the amino acid sequences of the selected neighbours. The second part shows the secondary structure assignments by DSSP (H/h: helix, E/e: strand, L/l: coil). The most frequent amino acid type is coloured in each column.

References

1. Duplessis, S. *et al.* Melampsora larici-populina transcript profiling during germination and timecourse infection of poplar leaves reveals dynamic expression patterns associated with virulence and biotrophy. *Mol. Plant Microbe Interact.* **24**, 808–818 (2011).
2. Joly, D. L., Feau, N., Tanguay, P. & Hamelin, R. C. Comparative analysis of secreted protein evolution using expressed sequence tags from four poplar leaf rusts (Melampsora spp.). *BMC Genomics* **11**, 422 (2010).
3. Hacquard, S. *et al.* A comprehensive analysis of genes encoding small secreted proteins identifies candidate effectors in Melampsora larici-populina (poplar leaf rust). *Mol. Plant Microbe Interact.* **25**, 279–293 (2012).
4. Holm, L. & Rosenström, P. Dali server: conservation mapping in 3D. *Nucleic Acids Res.* **38**, W545-549 (2010).
